# Exploring the Animal Waste Resistome: The Spread of Antimicrobial Resistance Genes Through the Use of Livestock Manure

**DOI:** 10.3389/fmicb.2020.01416

**Published:** 2020-07-22

**Authors:** Alice Checcucci, Paolo Trevisi, Diana Luise, Monica Modesto, Sonia Blasioli, Ilaria Braschi, Paola Mattarelli

**Affiliations:** Department of Agricultural and Food Science, University of Bologna, Bologna, Italy

**Keywords:** veterinary antibiotics, animal manure, antibiotic resistance genes, crop soils, antimicrobial resistance

## Abstract

Antibiotic resistance is a public health problem of growing concern. Animal manure application to soil is considered to be a main cause of the propagation and dissemination of antibiotic residues, antibiotic-resistant bacteria (ARB), and antibiotic resistance genes (ARGs) in the soil-water system. In recent decades, studies on the impact of antibiotic-contaminated manure on soil microbiomes have increased exponentially, in particular for taxonomical diversity and ARGs’ diffusion. Antibiotic resistance genes are often located on mobile genetic elements (MGEs). Horizontal transfer of MGEs toward a broad range of bacteria (pathogens and human commensals included) has been identified as the main cause for their persistence and dissemination. Chemical and bio-sanitizing treatments reduce the antibiotic load and ARB. Nevertheless, effects of these treatments on the persistence of resistance genes must be carefully considered. This review analyzed the most recent research on antibiotic and ARG environmental dissemination conveyed by livestock waste. Strategies to control ARG dissemination and antibiotic persistence were reviewed with the aim to identify methods for monitoring DNA transferability and environmental conditions promoting such diffusion.

## Introduction

In recent decades, the overuse and misuse of antibiotics in human and veterinary medicine has become a serious public health issue (World Health Organization, 2014; Aidara-Kane et al., 2018). The increased number of resistant pathogens and commensal bacteria has been associated with the environmental spread of antibiotics and the propagation of antimicrobial resistant genes (ARGs; Levy, 1998; Witte, 1998; He et al., 2020). Furthermore, the environmental diffusion of antibiotics may lead to the change (Han et al., 2018) and loss (Chen et al., 2019) of microbial community diversity in soil (Kemper, 2008).

Antibiotics are used worldwide in livestock production, thus increasing the risk of antimicrobial resistance (AMR) spread. When administered for prophylactic treatments, antibiotics can directly increase selective pressure, thus favoring the generation of antibiotic-resistant bacteria (ARB; Pruden et al., 2013; Troiano et al., 2018; Blau et al., 2019). For these reasons, improved livestock and waste management strategies (i.e., diets, proximity between animals, waste treatment, use of additives, and operating conditions) should be adopted to limit the use of antibiotics in animal husbandry.

**FIGURE 1:**
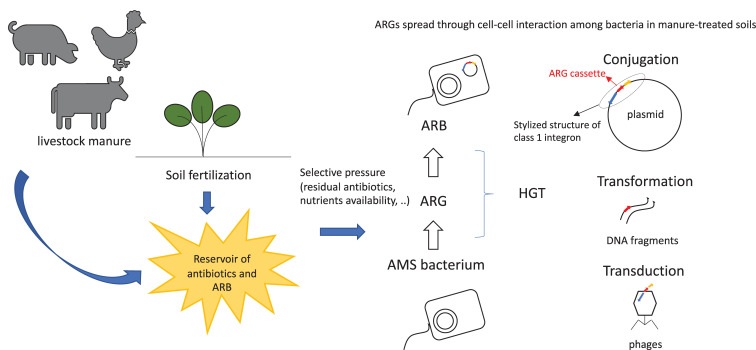
Spread of ARGs and ARB in farm-related environments. ARB, antimicrobial resistant bacteria; ARG, antibiotic resistance gene; AMS, antimicrobial sensitive; HGT, horizontal gene transfer.

Antimicrobial resistant genes can enter and persist in ecosystem through multiple pathways. They spread across soil (Binh et al., 2007), crops (Su et al., 2015), and gut microbial communities of wild and livestock animals and of humans (Yadav and Kapley, 2019). Antimicrobial resistant genes’ spread occurs through horizontal gene transfer (HGT) of mobile genetic elements (MGEs), as phages, plasmids (Fondi and Fani, 2010), transposons, or integron gene cassettes (Figure 1). The acquisition of AMR by bacteria may be due to spontaneous mutations (Woodford and Ellington, 2007) or, more frequently, by gaining specific ARGs from other bacteria through HGT. High density of microbial cells in the presence of antimicrobial compounds and nutrients, as observable in manure (Blau et al., 2018), triggers HGT events among bacteria, thus conferring selective advantage to the hosts (Thomas and Nielsen, 2005). Mutations are essential for the continuous evolution of ARGs, producing hundreds of variants which are hardly identifiable and increasingly dangerous for the environment (Woodford and Ellington, 2007).

In this review, the effect of antibiotic occurrence in animal manure on the dissemination of AMR and ARGs in agricultural fields are discussed in a critical way. The main strategies to mitigate ARGs’ dissemination and to control antibiotic persistence are also reported. Methods monitoring changes in microbial communities and transferability and environmental diffusion of DNA were addressed as well.

## The Dissemination Mechanisms of Environmental Resistome

The “resistome,” i.e., the total amount of resistance genes associated with an ecosystem (Finley et al., 2013), is generally mediated by conjugative plasmids. The resistome confers resistance of antibiotics and heavy metals to microorganisms, thus enhancing their survival in hostile environments (Bennett, 2008; Song et al., 2017). IncP-1, a common environmental plasmid group, is largely known for its efficient conjugative transferability potential and stable replication in a wide range of Gram-negative bacteria (Heuer et al., 2012). Conversely, plasmids IncF (Villa et al., 2010), IncI (Blau et al., 2018), and IncQ (Rawlings and Tietze, 2001) show a narrower host range. These plasmids are assumed to be important for the dissemination of ARG in *Escherichia coli* and other Enterobacteriaceae (Johnson and Nolan, 2009; Suzuki et al., 2010; Heuer et al., 2012; Van Houdt et al., 2013). As evidence, the study of the mechanisms of diffusion of these plasmids (Teuber, 2001) and compatibility evolution with broad or narrow host ranges should allow for ARG diffusion prediction.

Integrons play a key role in the fast spread of resistance determinants toward antibiotics. They are genetic elements composed of a gene encoding an integrase and an integration site for exogenous gene cassettes, which can be acquired and converted in functional and expressed genes (Mazel, 2006). Integrons can move horizontally in bacterial populations by frequent integration in plasmids or in transposons (Rowe-Magnus and Mazel, 2002). According to their aminoacidic sequence, integrases are divided into several classes. Classes 1, 2, and 3 (Inti1, Inti2, Inti3) were the first to be identified as associated with MGEs, while class 4 (Inti4) was associated with chromosomal integration (Deng et al., 2015). Among elements which facilitate DNA transfer, class 1 integron (*int1*) is the most frequently identified as responsible for spreading antibiotic resistance determinants amongst commensals and pathogens of humans and domesticated animals. Moreover, *int1* cassette was found in different environments, such as fresh water, sediments, and sludge (Collis and Hall, 1995; Hall and Collis, 1998; Nardelli et al., 2012; Borruso et al., 2016), where it showed significantly positive correlations with the relative ARG abundance (Zhao et al., 2019).

Antibiotic residues, once entered into soil through manure application, can enhance persistence and HGT of ARGs (Binh et al., 2007; Zhao et al., 2019) through plasmids and integrons (Gotz and Smalla, 1997; Smalla et al., 2000; Sengeløv et al., 2003), promoting the spread of ARB in the environment and affecting the microbial community composition (Chen et al., 2019). Although manure-derived bacteria cannot always adapt to new environments, the antimicrobials can favor the enrichment of specific bacterial taxa in soils (through positive selection) and suppress others (Ding et al., 2014). In addition, the concentration of antibiotics in manure, usually at a sub-inhibitory level, can affect the interactions among strains and impact on gene expression and regulation (Gillings, 2013; Jechalke et al., 2014; Brüssow, 2015).

When manure is used as a fertilizer for crop production, both the increased ARB load and the antibiotic residues contained within may have negative effects on plant development and food product quality (Verraes et al., 2013; Mirza et al., 2020; Muhammad et al., 2020). In addition, antibiotic residues can persist and accumulate in the environment (Jechalke et al., 2014) by adsorption on soil solid phases (Du and Liu, 2012).

## ARGs in the Environment

The majority of antibiotics are naturally produced by microbes as a self-protection mechanism against other microorganisms. ARGs have been always present in the environment. ARGs encoding resistance for a large set of antibiotics have been found in 30,000-year-old Beringian permafrost and in bacteria isolated from prehistoric caves (D’Costa et al., 2011; Berglund, 2015). When present in the environment at a sub-inhibitory concentration, antibiotics frequently play a role in transcription regulation and in the exchange of signals among cells (i.e., quorum sensing mechanism and conjugation) (Reygaert, 2018).

Antibiotic resistance consists of a large variety of mechanisms, such as inactivation by specific cleaving enzymes, exclusion from cells via efflux pumps, interference with protein synthesis, limitation of drug uptake, and modification of antibiotic target. Resistance acquired through MGEs and plasmids is responsible for the last two mechanisms in which the resistance extent depends on bacterial species and acquired ARGs (Reygaert, 2018; Kraemer et al., 2019). The antibiotic selective pressure driving the acquired resistance determines accurate ARGs’ specialization, thus making the environment a potential reservoir.

Anthropogenic activities affect antibiotic and ARGs’ spread with somewhat predictable effects (Vikesland et al., 2017). In livestock farming, the use of antibiotics varies depending on the farming type and location, having a considerable effect on ARGs’ concentration. Among the ARGs most frequently detected in livestock production, those related to sulfonamide resistance (*sul*) (Table 1) are particularly diffused in aquatic systems (Chen et al., 2015; Makowska et al., 2016). In surface and fresh waters, *sul* genes were found in IncQ plasmid group (Sköld, 2001; Berglund, 2015). Similarly, diaminopyrimidine genes (*dfr*), which confer resistance to antimicrobial trimethoprim, have been identified in both class 1 and class 2 integrons (Deng et al., 2015). Similarly, quinolone resistance *qnr* genes have been frequently associated with different plasmid groups. Both *dfr* and *qnr* genes easily disseminate in the environment, being found in surface waters (Berglund, 2015), wastewaters, and related irrigated soils (Dalkmann et al., 2012). Tetracycline resistance genes (*tet*) are widely diffused in different pathogenic and environmental bacteria (Roberts, 2005) and are often detected in sewage treatment plants, soil, and surface and ground water (Chee-Sanford et al., 2001; Berglund, 2015). In the same environments, *erm* genes, which are the most widespread macrolides resistance gene, were isolated.

**TABLE 1:** The most commonly used antibiotics and the relative ARGs in livestock production (DHPS, dihydropteroate synthase; DHPR, dihydropyridine-resistant).

**Antibiotic family**	**Most used**	**Animal Farming**	**Use**	**Contrasted bacteria and recognized main targets**	**Resistance mechanism**	**Main ARGs**
Macrolides	Tylosin	Cattle	Gastrointestinal and respiratory infections	Gram-positive bacteria.	Interference with protein synthesis (sequestration of mRNA ribosome-binding site)	*erm*, *msr*, *mef* genes
	Erytromycin	Pig		Main target: *Lawsonia intracellularis*		
	Clarithromycin	Poultry		*Staphylococcus aureus*		
Sulfonamides	Sulfamethazine	Cattle	Urinary tract infections	Gram-positive and Gram-negative bacteria. Main target: *Enterobacteriaceae, Pasteurellaceae*	Interference with folic acid synthesis competing for the enzyme DHPS	*sulI, sulII* genes
		Pig	Respiratory infections			
		Poultry				
Tetracyclines	Chlortetracycline	Cattle	Systemic and local infections	Gram-positive and Gram-negative bacteria	Interference with efflux pump systems	*tet* genes
	Oxytetracyclines	Pig	Gastrointestinal and respiratory infections			
	Doxycycline	Poultry				
Quinolones	Fluoroquinolones (Enrofloxacin, Danofloxacin, Marbofloxacine)	Pig	Intestinal infections	Gram-positive and Gram-negative bacteria, including mycobacteria, and anaerobes	Mutations in the genes encoding quinolone target DNA gyrase and topoisomerase IV, interference with efflux pump systems	*qnr* genes
		Cattle				
β-lactams	Penicillins (Amoxycilline, Ampicillines) Cephalosporins, Carbapenems	Pig	Respiratory diseases	Gram-positive and Gram-negative bacteria	Interference with cell wall synthesis and permeability, inactivation through β-Lactamase enzyme	*bla, amp, pen* genes,
		Cattle	Necrotic enteritis			
		Poultry				
		Dog				
		Cat				
Aminoglycosides	Streptomycin, Spectinomycin, Neomycin, Aspramycin, Gentamycin, Lincomycin	Pig	Intestinal infections	Gram-positive, and Gram-negative bacteria, if aerobic	Inhibition of protein synthesis (rhibosome interference)	*aac, aad*, *aad aph* genes
		Poultry				
Phenicols	Chloramphenicol	Pig	Respiratory disease, foot rot	Broad spectrum. Main target: *Photobacterium, Salmonella, E. coli*	Enzymatic modification of antibiotic molecules	*cat, pp-flo, flo* genes
	Thiamphenicols (thiamphenicol, florfenicol)					
Diaminopyrimidines	Trimethoprim	Horse	Post-weaning scours	Gram-positive and many Gram-negative bacteria. Main target: *Enterobacteriaceae*	Interference with folic acid synthesis by binding the enzyme DHFR	*dfr* genes
		Pig				
Polypeptides	Bacitracin, Colistin	Pig	Intestinal diseases	Gram-positive (Bacitracin) or Gram negative (Colistin) bacteria. Main Gram negative target: *E. coli Salmonela* spp. *Pseudomonas aeruginosa*, *Klebsiella pneumoniae*, or *Acinetobacter*.	LPS modification, efflux pump systems regulation	*pmr,pho, mcr, kpn* genes
		Poultry		Main Gram positive target: *Campylobacter*		
Lincosamides	Lincomycin	Pig	Respiratory and Intestinal infections	Gram positive bacteria, most anaerobic and some mycolpasma species. Main target: *Staphylococcus aureus*	Alteration of the antibiotic target site	*lnu, lin, erm* genes
		Poultry				
		Cattle				
Pleuromutilins	Tiamulin	Pig	Respiratory and Intestinal infections	*Pasteurellaceae, Brachyspira, Micoplasma*	Alteration/protection of the antibiotic target site	*vga, sal, lsa* genes
	Valnemulin	Poultry				

*References: (Schwarz et al., 2001; Petinaki et al., 2008; Guardabassi et al., 2009; Abbas et al., 2011; Van Hoek et al., 2011; Li et al., 2013; Shang et al., 2013; Tasho and Cho, 2016; Deng et al., 2017; Aghapour et al., 2019; https://www.msdvetmanual.com/pharmacology/antibacterial-agents).*

Essentially, ARGs’ diffusion is associated with a stress response activated by exposure to antibiotics as well as with the mobilization of several integrative and conjugative elements. ARGs’ maintenance depends on their considerably low fitness cost. In fact, once a specific ARG has been acquired by a bacterial cell, it must evolve to produce more benefits than costs in order for multiple copies of the same gene to be kept and to maintain the expression control of genes in MGEs (Bengtsson-Palme et al., 2017). Furthermore, as already mentioned, nutrient rich environments can positively influence the ARGs’ spread and facilitate cell–cell interactions (Manaia et al., 2018) (Figure 1).

## The Use of Veterinary Antibiotics

In veterinary medicine, antimicrobials can be used as therapeutics and/or growth promoters. Antibiotic growth promoters (AGPs) are antimicrobial substances administered at a sub-therapeutic dose for a prolonged time with the main purpose being to improve the feed conversion rate, especially in young animals, raising the economical profit of farmers. Since 2006, both the European Union and Australia have forbidden the use of AGPs. Nevertheless, in most other countries the use of AGPs is still permitted (Guardabassi et al., 2009).

Among breeding farms, poultry and pig livestock have received the majority of antibiotics for therapeutic or prophylactic use (Ungemach, 2000; Kim et al., 2011), resulting in an abundance of ARGs greater than three orders of magnitude compared to other farming systems, such as fish and cattle farming. Several studies confirmed swine farms as a hot-spot for ARB and ARGs (Rosen, 1995; Cromwell, 2002; de Greeff et al., 2019; Petrin et al., 2019). Recently, the scientific community investigated prevalence, abundance, and possible mobilization of ARGs in pig farms and surrounding environments (Hölzel et al., 2010; Marti Serrano, 2014; Petrin et al., 2019; Van den Meersche et al., 2019; Wu et al., 2019).

Table 1 summarizes the main antibiotic families and the most used antimicrobics in livestock animals for therapeutic use. Nowadays, more than 150 antimicrobial compounds in livestock production are used. The residues inevitably end up in the environment because of manure application on agricultural lands (Baguer et al., 2000). In 2010, more than 63,000 tons of antimicrobials were consumed by livestock across the globe. The predicted growth of the world’s population allows for an estimated increase in antibiotic consumption of up to 105,000 tons by 2030 (Tasho and Cho, 2016). For this reason, specific action plans have been defined to reduce the use of antibiotics as therapeutics for livestock in several countries (i.e., the European One Health Action Plan against Antimicrobial Resistance, 2017; the National Strategy to Combat Antibiotic-Resistant Bacteria, proposed by the White House, 2014; the National Action Plan to Contain Antimicrobial Resistance issued by the Chinese National Health and Family Planning Commission, 2016–2020).

## Manure Treatments

Besides direct collection into aerobic or anaerobic lagoons, animal manure can undergo drying and liquid-solid phase separation. Manure solid phase, as well as whole manure if shovellable, is traditionally composted to produce biofertilizer. Currently, anaerobic digestion and biological treatments of animal manure are often adopted on intensive animal farms (Van Epps and Blaney, 2016).

Composting can substantially reduce the antibiotic load, especially during the thermophilic phase (Zhang et al., 2019), but recalcitrant antibiotics accumulate in compost products and in amended soil (Bohrer et al., 2019; Zang et al., 2019). A general ARG abatement (0.7–2.0 log decrease) is obtained through thermophilic composting of swine, cattle, and poultry manure, depending on manure type and operational conditions (He et al., 2020).

Biological treatments of animal manure and wastewater, which are adopted to reduce the environmental input of nitrates, slightly decreases the levels of antibiotic residues and pathogenic bacteria (Van den Meersche et al., 2019). Antimicrobial resistant gene reduction of 0.1–3.3 log is observed in swine manure after treatment (He et al., 2020).

Anerobic digestion (AD) is adopted to stabilize manure with a final production of methane (Fubin et al., 2016, 2017). A 0.3–52 log decrease of ARGs was observed in digestate from swine wastewater (He et al., 2020). Interestingly, the higher the content of volatile solids in manure and the mixing rate, the higher the ARGs number in the digestate (Turker et al., 2018). The combined pasteurization and AD of swine manure reduced sole archaeal communities, whereas simple AD affected bacteria and archea (Fubin et al., 2020). Manure pretreatment with bacterial strains is effective in degrading antibiotics (Liu et al., 2019) and enhancing biogas production, but the overall effect on ARB and ARGs was not addressed.

Constructed wetlands are vegetated aquatic systems that can be adopted for the treatment of wastewater and agricultural drainage water (Lavrnic et al., 2018). Their ability to reduce ARGs in swine wastewater resulted in a 0.18–3 log decrease (He et al., 2020).

Oxidizing post-treatments, as ozonation or Fenton conditions, can be used on animal or treated wastewaters to degrade antibiotics and bacteria thanks to the activity of reactive oxygen species (Balcıoğlu and Ötker, 2003; Ikehata et al., 2006; Uslu and Balcıoğlu, 2009). Among advanced oxidation processes, highly costly ionizing radiations are known for their ability to destroy microbial DNA. Therefore, affordable combinations of ionizing radiation and oxidation allows for the degradation of antibiotics and ARGs in organic matrices, although with a high biological and environmental risk (Chu et al., 2019, 2020).

## Different Approaches to Resistome Profiling Study

Even though AMRs introduced in the environment with animal manure have been largely explored (Dolliver et al., 2008; Selvam et al., 2012b), contradictory information exists regarding the fate of ARGs (Selvam et al., 2012a; Wang et al., 2015; Xie et al., 2016). The growing need for the control of ARGs’ spread prompted the scientific community to set up and to validate refined molecular methods for the study of ARGs’ dissemination dynamics among environmental microbial communities.

Both 16S rRNA amplicon and untargeted sequencing can be considered exhaustive methods for the exploration of microbial community structure in manure-fertilized soil and farm waste. Several studies on resistome diffusion in wastewater treatment plants (Yadav and Kapley, 2019), sewage sludge composting units (Su et al., 2015), and urban sewage support the metagenomic approach (Hendriksen et al., 2019) in monitoring ARGs’ level during treatments and seasonal changes. A recent work (Han et al., 2018) showed that the shift in soil bacterial communities caused by manure application leads to changes in the soil bacteria resistome.

Recently, studies on the detection of genetic markers associated with AMR (transposases and class 1 integron-integrase genes) and ARGs have been markedly increasing. The quantification of ARGs in soils amended with livestock and swine manure (Brooks et al., 2014; Tao et al., 2014) was performed with high-throughput qPCR assay (Rocha et al., 2018; Blau et al., 2019). In a recent study, both intracellular and extracellular DNA containing ARGs were quantified in sludge at about 10^10^ and 10^12^ copies per gram, respectively (Dong et al., 2019). Here, the intracellular ARGs were assessed through conjugation with cell-cell contact, whereas the extracellular ARGs were assessed through natural transformation. Several works on different manure types focused on the quantification of targeted genes *intI1* and *intI2* for class 1 and 2 integron-integrase genes and *korB* gene, specific for IncP-1 plasmids, together with ARGs (Hu et al., 2016; Blau et al., 2018, 2019).

As already reported, plasmid-mediated ARGs’ diffusion is frequently used, especially for the role of plasmids in the rapid bacterial adaptation and fitness improvement (Smalla et al., 2000). Exogenous plasmid isolation techniques (Bale et al., 1988) clarified how plasmids diffuse in different environments. Recently, plasmids from municipal sewage sludge and recipient bacteria were analyzed for their transferability by exogenous isolation (Blau et al., 2018; Wolters et al., 2018). Referring to pig manure samples, four IncQ-like plasmids were isolated in recipient strains: *Pseudomonas putida* UWC1, *Acinetobacter* sp., *Ralstonia eutropha*, *Agrobacterium tumefaciens*, and *E. coli.* The plasmid transferability in *E. coli* strains was not efficient, underlying a broad but highly specific host range (Smalla et al., 2000).

Recently, simplified mathematical models have been applied to predict and quantify ARGs’ spread in livestock animal gut microbiomes (Andersen et al., 2020) and in agricultural waste (Baker et al., 2016). In such environments, the variables involved in the ARGs’ spread are countless and depend on a wide range of intrinsic and extrinsic factors, such as genetic mechanisms of ARB replication, HGT dynamics, environmental and stressor conditions, and microbiota composition. Therefore, future research should focus on the improvement of predictive models of ARGs’ dissemination mechanism, exploitable for targeted operations in livestock waste management.

## Conclusion

Although a decrease in the use of antibiotics in livestock production is highly recommended, antibiotics’ overuse remains an important issue to solve. The uncontrolled spread of ARB and ARGs in the environment due to soil manuring is of serious concern. Many studies highlight ARGs’ presence in microbial communities of livestock manure and manured agricultural fields, despite the improved livestock and waste management strategies to contain in-farm ARGs’ spread. In the last thirty years, knowledge on pathways of ARGs’ diffusion from animal waste to the environment was enriched by multidisciplinary research approaches.

In light of the current knowledge, the study of the dynamics of AMR and ARGs’ spread in manure and environments surrounding livestock farms should combine molecular and functional genetics strategies with prediction models of the diffusion of MGEs (integrons and plasmids) and metagenomic data.

## Author Contributions

AC: original draft preparation, figure and table conceptualization, review, and editing. PT, MM, and SB: review. DL: original draft preparation and table preparation. IB and PM: original draft preparation and review. All authors contributed to critically revising the manuscript and gave final approval for publication.

## Conflict of Interest

The authors declare that the research was conducted in the absence of any commercial or financial relationships that could be construed as a potential conflict of interest.
